# Influence of Manufacturing Regimes on the Phase Transformation of Dental Zirconia

**DOI:** 10.3390/ma14174980

**Published:** 2021-08-31

**Authors:** Markus Wertz, Hieronymus Hoelzig, Gert Kloess, Sebastian Hahnel, Andreas Koenig

**Affiliations:** 1 Department of Prosthodontics and Material Sciences, Leipzig University, 04103 Leipzig, Germany; markus.wertz@medizin.uni-leipzig.de (M.W.); sebastian.hahnel@medizin.uni-leipzig.de (S.H.); 2 Institute of Mineralogy, Crystallography and Materials Science, Leipzig University, 04103 Leipzig, Germany; hieronymus.hoelzig@uni-leipzig.de (H.H.); kloess@uni-leipzig.de (G.K.)

**Keywords:** yttria-stabilized zirconia, X-ray diffraction, Rietveld refinement, fixed dental prosthesis, rhombohedral phase, computer-assisted manufacturing

## Abstract

Background: The influence of typical manufacturing regimes for producing fixed dental prostheses (FDPs) from yttria partly-stabilized zirconia polycrystals (3Y/4Y/5Y-TZP) on the phase composition is quantified. Methods: Fixed dental prostheses (FDPs) were designed using a CAD process and machined from different Y-TZP blanks from two manufacturers differing in yttria contents. Subsequent to sintering, the FDPs were glaze fired and air-blasted using alumina particles. Phase composition was determined with X-ray diffraction and quantified with Rietveld refinement. Results: The blanks from VITA Zahnfabrik (VITA YZ HT, VITA YZ ST, VITA YZ XT) and Dental Direct (DD Bio ZX^2^, DD cube ONE, DD cube X^2^) featured a rhombohedral portion with rather small crystallites and a small monoclinic portion for 3Y/4Y-TZPs, which increased after machining and disappeared after sintering. Glaze firing and air-blasting with alumina particles had no significant influence on the phase composition. Conclusion: The phase history of dental zirconia is revealed, which may have implications on further processing and aging of the FDP (e.g. low temperature degradation) in mouth.

## 1. Introduction

**Figure 1:**
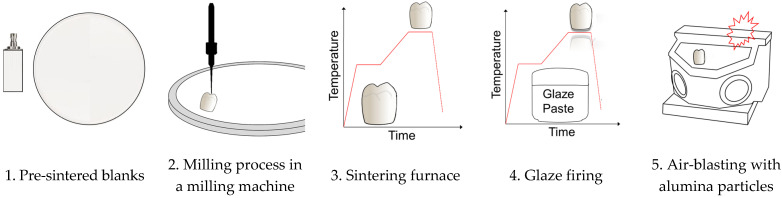
Steps in the process of fabricating a fixed dental prosthesis (FDP) from pre-sintered Y-TZP blanks in a dental laboratory.

Yttria-stabilized tetragonal zirconia polycrystalline (Y-TZP) ceramics were introduced in dentistry in 1998 as a material that is processed by CAD/CAM techniques [1]. Because of its outstanding mechanical (flexural strength between 750 and 1300 MPa [2,3]) as well as favorable physical [4,5,6,7] and aesthetic properties it is widely used for the fabrication of fixed-dental prostheses (FDPs). Today, zirconia is used in many dental applications such as fixed partial dentures (FPD), primary telescopic crowns, citations for Figure 1 in Section 1 dental implants, and suprastructures [8]. For the fabrication of esthetic restorations, a high translucency [9] of Y-TZP is necessary. This is most commonly achieved by inducing the isotropic cubic phase through an increased content of stabilizing Yttria (Y_2_O_3_), where less light scattering at the grain boundaries occurs. This procedure, however, coincides with a decrease in strength and toughness [10].

In order to ensure sufficient mechanical stability of the Y-TZP blanks (Figure 1, step 1) during the subtractive manufacturing process in the milling machine (Figure 1, step 2), the interfacial bonding in the ZrO_2_ powder is increased by hot isostatic pressing (hipping) and sintering under industrial conditions. In Y-TZP, typical hipping conditions feature a temperature of 1500 °C and a pressure of 200 MPa [11], which produce a mixture of monoclinic, rhombohedral, tetragonal, and cubic phases [12].

The ambient pressure phases of zirconia are monoclinic baddeleyite (M; P2_1_/c), which is stable up to 1205 °C for unstabilized zirconia, three tetragonal (T/T’/T”) phases (P4_2_/nmc) of which the tetragonal phase t is stable from 1205 °C to 2377 °C, and the cubic (C) fluorite structure (Fm$\bar{3}$m) which is stable from 2377 °C to the melting point [13,14,15]. In addition, there are also two orthorhombic (O/O’; Pbca & Pnam) high-pressure phases [14,16] and a rhombohedral/trigonal (R) phase (R$\bar{3}$), which may occur under mechanical stress and may be induced by hipping regimes [12,17,18,19].

While in the tetragonal and the cubic phases each zirconium ion is bonded to eight oxygen atoms, the zirconium atoms in the monoclinic and rhombohedral phases are only bonded to seven and six oxygen atoms [12,14]. This leads to a distortion of the elementary cell, which may result, e.g., in micro cracks and a degradation of the mechanical properties [14,20].

Doping with trivalent yttrium ions stabilizes the tetragonal and cubic phases through formation of oxygen vacancies [21]. A stabilization mechanism for tetragonal zirconia only is the ferroelastic domain switch: Tetragonal zirconia may be viewed as a layer structure with two kinds of Zr-O bonding: Zr-O_1_ (2.10 Å) is the stronger bonding within and Zr-O_2_ (2.34 Å) the weaker bonding between the layers [21]. It may change the preferred orientation of its domain structure, which may serve as an explanation for its high toughness [1].

In the dental laboratory, Y-TZP is processed in milling machines using industrially pre-sintered and baked block- or disc-shaped blanks. This process minimizes wear of the milling machine and its associated components, and increases the processing speed. The milling process (CAM) includes several steps, which may induce micro cracks [22] and eventually lead to grain detachment with consequent degradation in strength. An increase in monoclinic phase fraction but no rhombohedral phase fraction have been identified after milling [23].

Subsequent to milling, the restorations are sintered (Figure 1, step 3) in air at a maximum temperature of 1450 °C or 1530 °C (sintering instructions of the manufacturers VITA/Dental Direct). Sintering causes a volume reduction, a decrease in roughness and pore volume (ref. [24,25,26]), and the transformation from the monoclinic to the tetragonal phase [27]. In order to achieve a complete phase transformation and, at the same time, minimize restraint stresses in the cross-section of the restoration, the heating and cooling rate [28] as well as the holding time during the re-sintering process have to be controlled.

Finally, the outer surface of the restoration is glazed and fired (Figure 1, step 4), while the inner surface of the restorations are subjected to air-blasting using alumina particles [19] (Figure 1, step 5). Glaze firing (Figure 1, step 4) decreases the flexural strength of Y-TZP [29], while air-blasting with alumina particles increases the surface roughness and reduces flexural and compressive strength [30]. Air-blasting may induce a transformation of tetragonal and cubic phase fractions into a rhombohedral (trigonal) phase [19].

The current study investigates phase transformations in various Y-TZPs during the entire fabrication process of FDPs and aims to elucidate how far fabrication processes affect the properties of Y-TZPs on a molecular level. This might be useful to improve the clinical performance of the materials, to get a better understanding for phenomena like low temperature degradation (LTD) [31]. The null hypothesis of the current study is that the fraction of the monoclinic phase increases during the fabrication process of a FDP.

## 2. Materials and Methods

### 2.1. Materials

Six Y-TZP blanks from two manufacturers (VITA Zahnfabrik (Vita Zahnfabrik H. Rauter GmbH & Co. KG, DE-79704 Bad Säckingen, Germany; VT) and Dental Direkt (Dental Direkt GmbH, DE-32139 Sprenge, Germany; DD)) with three different yttria contents were analyzed (Table 1).

These six blanks (Figure 1: step 1) were processed (CAD/CAM) using the CAD software ceramill mind 2.4 7437 (Amann Girrbach AG, AT-6842 Koblach, Austria) and an inLab MC X5 (Dentsply Sitrona Deutschland GmbH, DE-64625 Bensheim, Germany) milling machine. For each step of the fabrication process, one single crown FDP was prepared from each Y-TZP. An upper premolar (wall thickness: buccal/palatinal 2.496 mm; mesial 0.657; distal: 0.690) was used as a template. For simplifying the measurements, the FDPs had no occlusal cusps (Figure 1: step 2).

The sintering process (Figure 1: step 3/Table 2) was performed in accordance with the individual instructions issued by the two Y-TZP manufacturers using a conventional sintering furnace for zirconia (VITA Zyrcomat 6000 MS, Vita Zahnfabrik H. Rauter GmbH & Co. KG, Germany).

Glaze firing (Figure 1: step 4/Table 3) was performed in a conventional furnace (VITA Vacumat 6000 M, Vita Zahnfabrik H. Rauter GmbH & Co. KG, DE-79704 Bad Säckingen, Germany; VT). The glaze paste was prepared from VITA Akzent Plus Glaze LT and VITA plus Powder Fluid according to the instructions of the manufacturer (Table 3).

The FDPs were air-blasted (Figure 1: step 5/Table 2) with 50 µm diameter alumina particles and a maximum pressure of 2 bar on the outside, because of the XRD measuring arrangement. The air-blasted FDPs were not glaze fired.

### 2.2. Methods

#### 2.2.1. X-ray Diffraction

**Figure 2:**
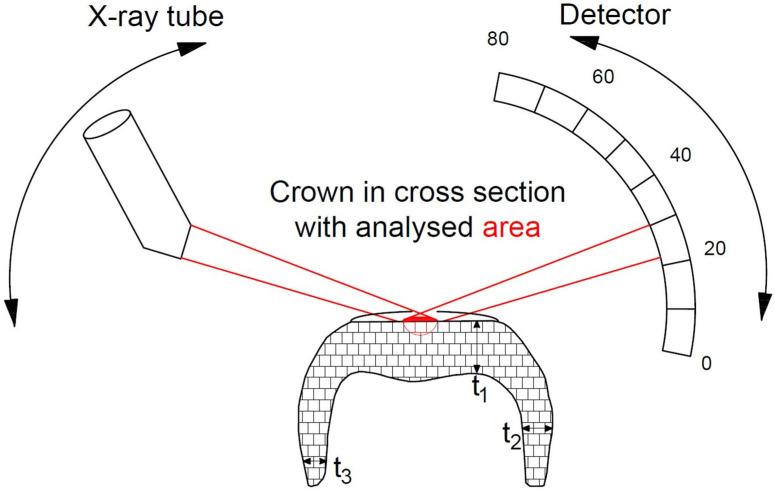
XRD measurement setup (Bragg-Brentano geometry) with the centrally arranged crown fabricated from Y-TZP with plane occlusal surface (t_1,max_~1.9 mm; t_2,max_~2.5 mm; t_3,max_~1.1 mm) and with the rotating X-ray source as well as the rotating detector; the red lines represent the X-ray beam diffracted by the sample with the local measurement point (ellipse).

A Bruker D8 discover (Bruker AXS Advanced X-ray Solutions GmbH, Karlsruhe) with a VÅNTEC-500 (Vantec Thermal Technologies, Fremont, CA, USA) as area detector and CuK_α_ radiation (1.54 Å, 40 mA, 40 kV) was used for the measurements. The goniometer radius on the secondary side was 300 mm (Figure 2).

The integration of the measured curves (Appendix A) was carried out with the program DIFFRAC.EVA (Version 3.1; Bruker AXS Advanced X-ray Solutions GmbH, Karlsruhe, Germany). The software TOPAS 4.2 (Bruker AXS Advanced X-ray Solutions GmbH, Karlsruhe, Germany) was used for Rietveld refinement.

The method of Garvie and Nicholson [32] with the correction of Toroya [33] for the determination of the monoclinic phase fraction (M) was discarded in favor of the far more accurate Rietveld method [34].

#### 2.2.2. Rietveld Refinement

**Figure 3:**
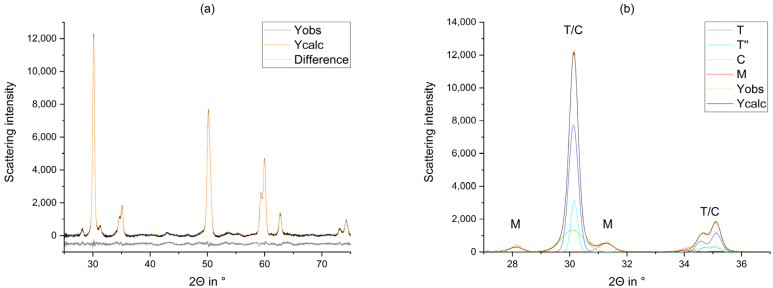
Comparison of the observed graph (Yobs), the refined graph (Ycalc), their difference (Diff), and the calculated curves (M, T, T”, C) of the machined FDPs fabricated from 3Y_DD_2 (**a**) 25°–75° (overview) (**b**) 27°–37° (main monoclinic reflections).

Structural data were gathered from literature [13,20,35,36,37] (Figure 3). Since dental FDPs are fabricated from sintered polycrystals, some of the processing steps (e.g., the air-blasting [19] cause an increase in surface roughness, which was compensated with a surface roughness correction according to Pitschke et al. [38]. Moreover, some processing steps caused strong texture effects, which were taken into account using a preferred orientation approach in accordance with March-Dollase [39,40].

There are six phases (three tetragonal phases, a monoclinic, rhombohedral/trigonal and a cubic phase) in yttria-doped zirconia [13,15,36]. However, we used only five phases (M, R, T, T”, C) for our refinement (cf. 4. discussion).

## 3. Results

**Figure 4:**
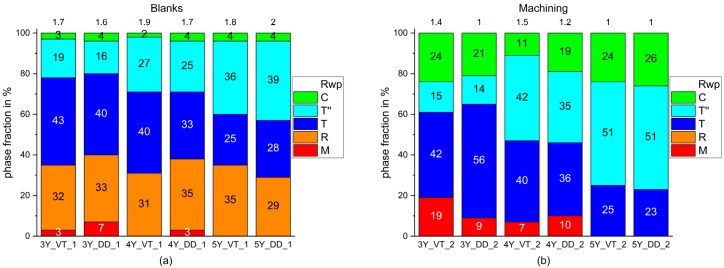
Phase fractions identified in the six different Y-TZPs prior to (**a**) and after milling (**b**).

Figure 4a displays the initial phase composition prior to processing. In contrast to the other process steps that had been analyzed, a relevant amount of rhombohedral (trigonal) phase (R$\bar{3}$) was identified. In addition, there were small monoclinic and cubic fractions in samples with lower (3Y/4Y) yttria content, which increased after milling (Figure 4b).

**Figure 5:**
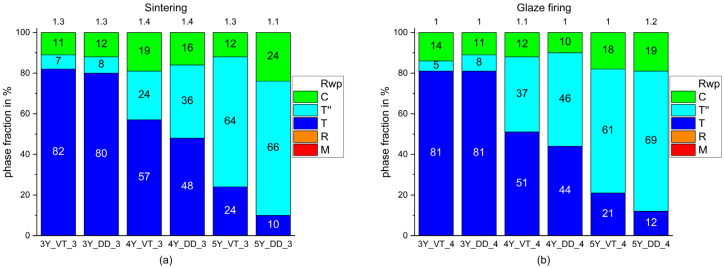
Phase fractions identified in the six different Y-TZPs after sintering (**a**) and glaze firing (**b**).

The monoclinic phase fraction disappeared after sintering (Figure 5a), while the fraction of the tetragonal phase T’’ and the cubic phase decreased.

Glaze firing (Figure 5b) produced only little changes in the phase fractions that were within the tolerance range.

**Figure 6:**
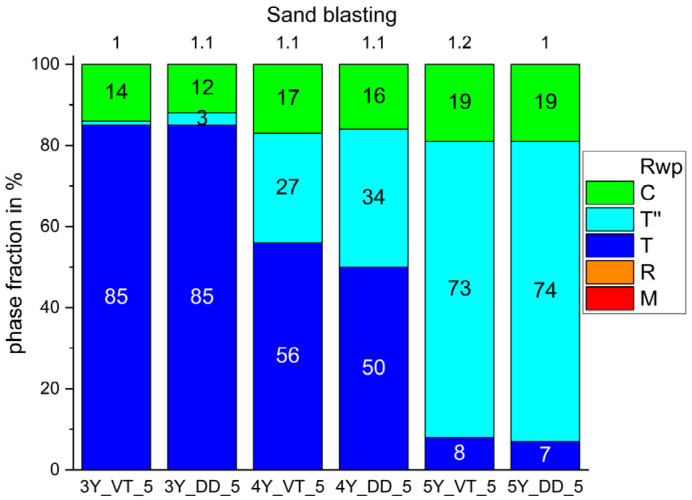
Phase fractions identified in the six different Y-TZPs after air-blasting with alumina particles.

Figure 6 displays a small increase in the tetragonal phase t for 3Y-TZP and in the tetragonal phase T” for 5Y-TZP after air-blasting with alumina particles.

As expected, the fractions of the cubic (c) and the tetragonal phase T’’ increased with higher yttria contents.

## 4. Discussion

**Figure 7:**
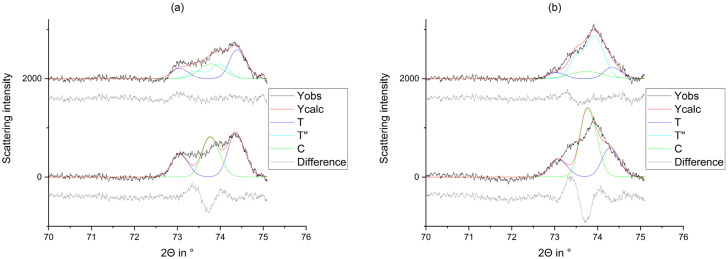
Comparison of the observed graph (Yobs), the refined graph (Ycalc), and the calculated curves (M, T, T”, C) of the machined FDP fabricated from (**a**) 3Y_DD_3 and (**b**) 5Y_DD_3 from 70°–75°. Above: The refinement with the tetragonal phase T”. Below: The refinement without the tetragonal phase T”.

Since the phases T” and C are difficult to distinguish, the model without the tetragonal phase T” is widely used in dentistry. However, we have included the tetragonal phase T” into our refinement, because the adjustment to the observed curve is far better and several publications [13,15,36] clearly show the existence of the phase. The best distinction allows the reflections around 74° (Figure 7) [41].

Some works also use models with the phase T’ [13,15,34,41], however, the tetragonal phase T’ predominantly exists at high temperatures [42] and its inclusion hardly improved the refinement.

**Figure 8:**
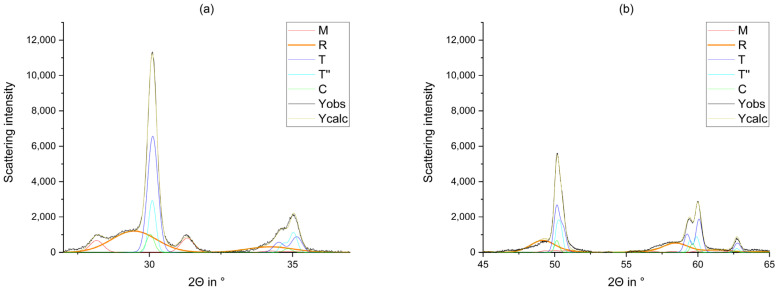
Observed curve, calculated curve, and calculated phases of the blank 3Y_VT_1. The rhombohedral phase is highlighted. (**a**) 27°–37° (**b**) 45°–65°.

Compared to the tetragonal, cubic, and monoclinic phases, the rhombohedral phase identified in the original blanks (step 1) featured much flatter reflections (Figure 8). This broadening results from the small crystallite size around 5 nm compared to 20–40 nm (taken from Rietveld Refinement) identified in the other phases in all samples. Kern et al. measured the crystallite size of the tetragonal phase with 18–27 nm [43].

Kitano et al. [12] reported that the rhombohedral phase is formed as a result from hipping procedures. Since the rhombohedral reflections were very broad, it would also be possible to interpret them as X-ray amorphous.

As expected, the fraction of the monoclinic phase increased after milling (step 2; Figure 4b) and diminished with sintering (step 3; Figure 5a). Neither glaze firing nor air-blasting with the selected conditions (Al_2_O_3_ particles, Ø 50 µm, 2 bar; Figure 6) resulted in a reformation of the monoclinic phase. The monoclinic phase is known for high hardness, brittleness, and very low translucency [44].

The parallel increase in the cubic phase may be a caused by the different ability to solve yttria: While the monoclinic phase solves few yttria, the cubic phase may contain great amounts of yttria [15].

**Figure 9:**
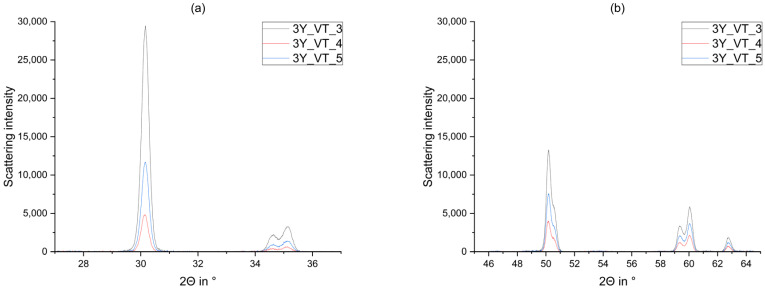
Comparison of the observed curves of 3Y_VT_3-5 after sintering (3), glaze firing (4), and air-blasting with alumina particles (5). (**a**) 27°–37° (**b**) 45°–65°.

Glaze firing (step 4; Figure 5b) and air-blasting (step 5; Figure 6)) caused no relevant phase transformation but a massive deterioration of the signal to noise ratio, which was probably due to the glaze layer (Figure 9) and an increase in surface roughness as a result of air-blasting [43]. The phase fractions identified for the different phases in the fabrication steps 3–5 were similar to the results published in other studies [2,31,34].

The Y-TZP samples from the manufacturer DD had a tendency towards phases with lower tetragonality (T”, C) than those manufactured by VT. It can be suspected that Y-TZPs from the manufacturers differ, for instance, in crystallite size. Smaller crystallite sizes such as identified in Y-TZP from VITA Zahnfabrik prefer phases with higher symmetry [45].

Low temperature degeneration (caused by thermal [4,31,46] or mechanical [5,47] stress and fostered by humidity [31]) of Y-TZP through a phase transformation from one of the tetragonal phases to the monoclinic phase is postulated [31] to be a major concern for dental applications of Y-TZP.

However, the monoclinic phase transformation caused by computer-assisted machining transforms into a tetragonal phase by sintering for all specimen of all manufacturers and yttria concentrations.

Therefore, no LTD is likely to take place during the CAD/CAM-process. However, as the monoclinic phase transformation is accompanied by the formation of zirconia domains with different yttria contents [13,15], it is conceivable that this may promote the retransformation to the monoclinic phase (low yttria solubility [15]).

## 5. Conclusions

We cannot confirm the null hypothesis, yet the following conclusions can be drawn:

1. The phase composition of the blanks feature a proportion of a rhombohedral phase fraction with very small crystallites.
2. The monoclinic phase fraction increases massively after milling and diminishes with the sintering for both the 3Y- and 4Y-specimens. The cubic phase fraction simultaneously increases.
3. Glaze firing and alumina-particle air-blasting cause little to no changes in phase composition. The signal to noise ratio decreases as a result of the glaze layer and increasing surface roughness.
4. The tetragonal phase is highly dependent on the yttria content. 3Y-TZPs prefer the tetragonal phase T and 5Y-TZPs prefer the tetragonal phase T”. The 4Y-TZPs prefer both phases, with a slightly higher proportion of the tetragonal phase T.

Further studies are necessary to clarify the impact of the above observations on the clinical performance of Y-TZP FDPs.

## Figures and Tables

**Table 1:** Y-TZP blanks analyzed in the current study (Dental Direct GmbH (DD), VITA Zahnfabrik (VT)).

Abbreviation	Product	Manufacturer	Yttria Content (mol%)	Flexural Strength (MPa) ^1^
3Y_VT	VITA YZ HT	VT	3	1200
3Y_DD	DD Bio ZX^2^	DD	3	1250
4Y_VT	VITA YZ ST	VT	4	>850
4Y_DD	DD cube ONE	DD	4	>1250
5Y_VT	VITA YZ XT	VT	5	>600
5Y_DD	DD cubeX^2^	DD	5	>750

^1^ According to the manufacturer.

**Table 2:** Sintering programs employed for processing the various Y-TZP blanks.

**Dental Direct**	**Sintering Program**
1	Heating up to 900 °C with 8 K/min
2	Dwell at 900 °C for 30 min
3	Heating up to 1450 °C (1530 °C for DD cube ONE (4Y_DD))
4	Dwell at 1450 °C (1530 °C) for 120 min
5	Cooling to 200 °C with 10 K/min
**VITA**	**Sintering Program**
1	Heating up to 1430 °C (1530 °C for VT YZ ST (4Y_VT))
	with 17 K/min for VT YZ HT (3Y_VT)), with 8 K/min for VT YZ ST (4Y_VT)) or with 4 K/min for VT YZ XT (5Y_VT))
2	Dwell at 1450 °C (1530 °C for VT YZ ST (4Y_VT)) for 120 min
3	Cooling to 200 °C

**Table 3:** Employed glaze firing program.

	Glaze Firing Program
1	Heating up to 500 °C
2	Heating up to 900 °C with 80 K/min
3	Dwell at 900 °C for 1 min
4	Cooling to 600 °C

**Figure A1:**
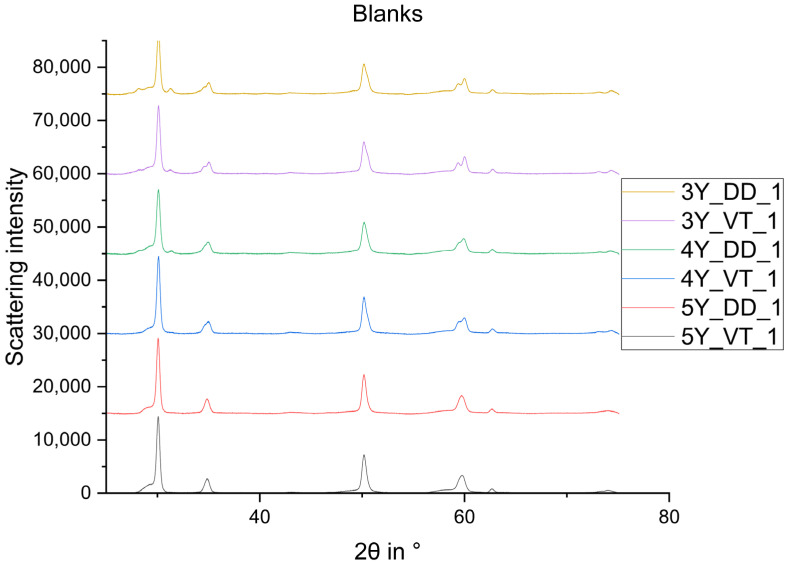

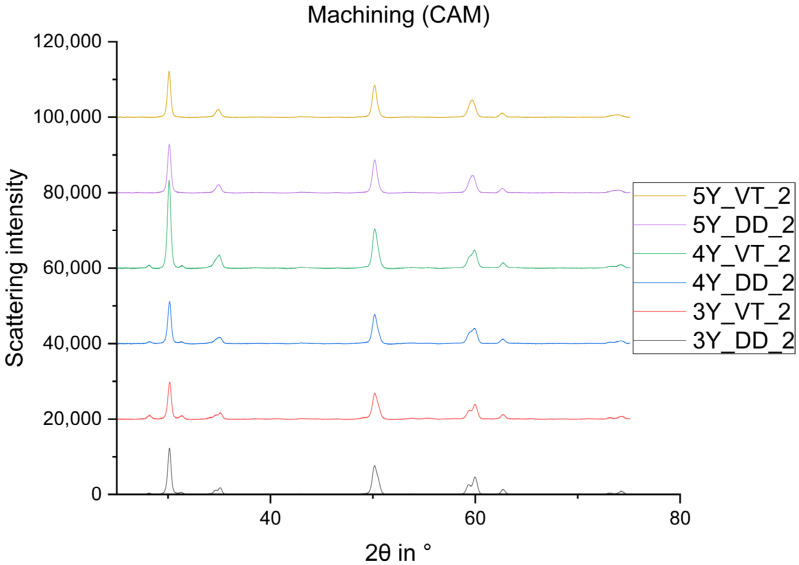
Observed curves of the blanks and the machined FDPs.

**Figure A2:**
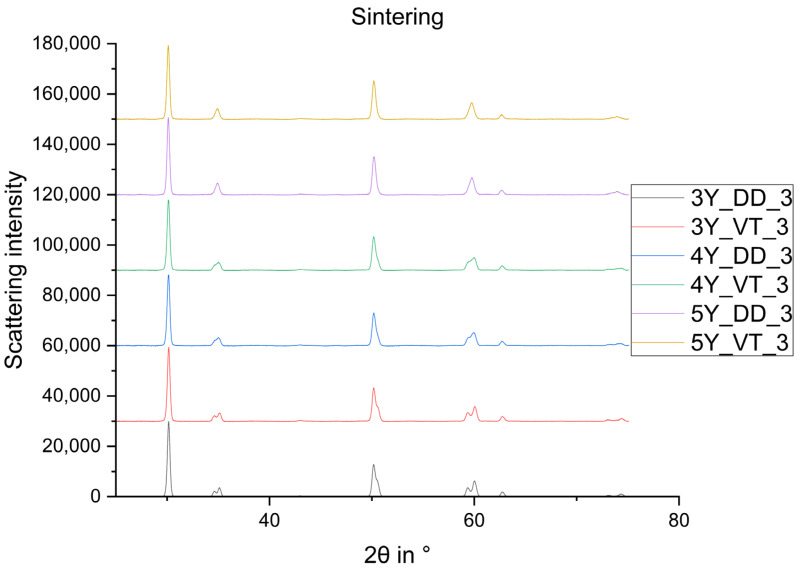

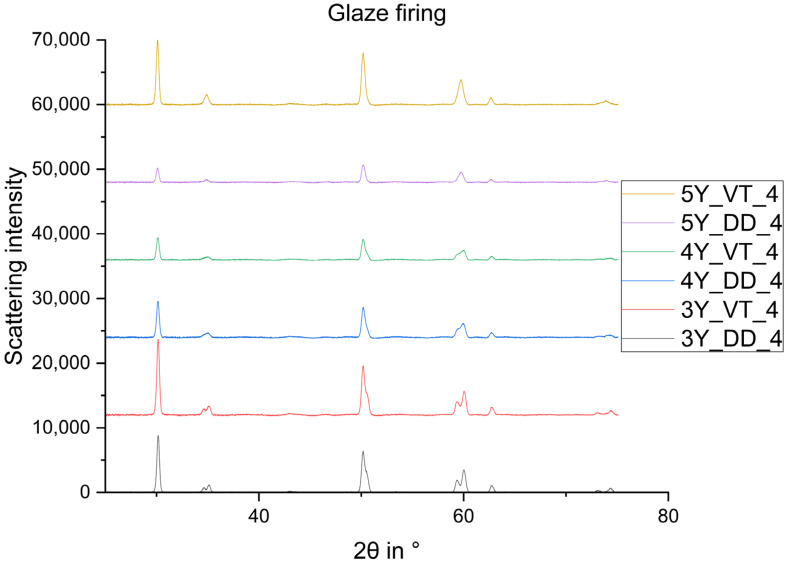
Observed curves of the sintered and the glaze fired FDPs.

**Figure A3:**
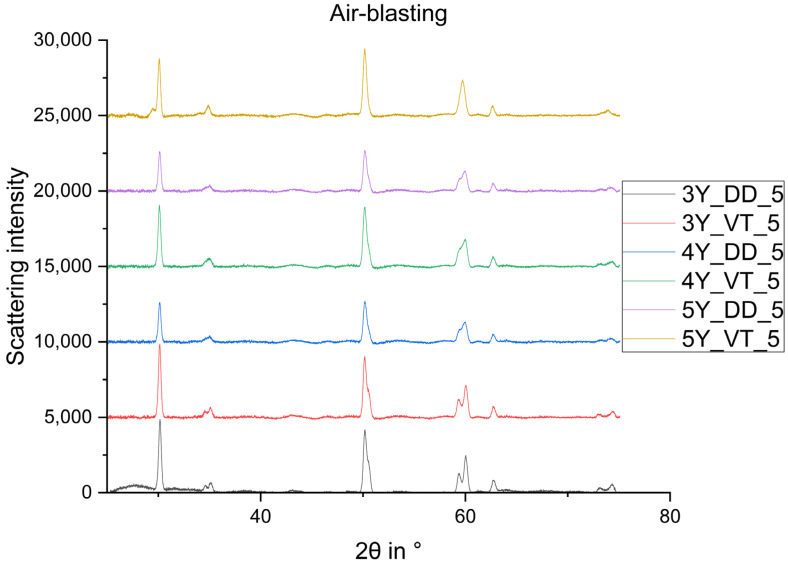
Observed curves of the air-blasted FDPs.

## Data Availability

The authors state that no external data were used.
